# Aerobic and muscle-strengthening guideline adherence and mobility limitation among adults with arthritis in the United States: a pooled cross-sectional analysis, 2020–2024

**DOI:** 10.1016/j.pmedr.2026.103692

**Published:** 2026-10-29

**Authors:** Dejin Wang, Jueliang Tian, Yuxi Peng, Shaoxue Wu, Yuhuan Feng

**Affiliations:** a School of Exercise and Health, Medicine & Technology College of Zunyi Medical University, No. 12, Yuanqu 1st Road, Xinpu New District, Zunyi, Guizhou 563006, China; b Doctoral Program in Physical Education, Graduate School, Universitas Negeri Jakarta, Jakarta 13220, Indonesia; c Doctoral Program in Educational Management, Graduate School, Universitas Negeri Jakarta, Jakarta 13220, Indonesia; d Ningxia Normal University, Guyuan, Ningxia 756099, China

**Keywords:** Arthritis, Aerobic activity, Muscle-strengthening activity, Mobility limitation, National Health Interview Survey, Complex survey analysis

## Abstract

**Objective:**

To compare mutually exclusive leisure-time aerobic and muscle-strengthening guideline-adherence patterns in relation to mobility limitation among U.S. adults with doctor-diagnosed arthritis.

**Methods:**

We pooled cross-sectional data from the 2020, 2022, and 2024 National Health Interview Survey in the United States for adults aged 18 years or older with doctor-diagnosed arthritis. Leisure-time activity was classified as neither guideline met, muscle-strengthening only, aerobic only, or both guidelines met. Survey-weighted modified Poisson regression estimated adjusted prevalence ratios for any difficulty walking or climbing steps, and marginal standardization estimated adjusted prevalences and absolute differences.

**Results:**

Among 22,344 adults, adjusted mobility-limitation prevalence was 53.8% for neither guideline, 50.3% for muscle-strengthening only, 39.9% for aerobic only, and 37.2% for both guidelines. Compared with neither guideline, meeting both guidelines was associated with lower prevalence (prevalence ratio, 0.62; 95% confidence interval, 0.58, 0.66) and an absolute difference of −16.6 percentage points. Aerobic-only adherence was also associated with lower prevalence, whereas the muscle-strengthening-only estimate was closer to 1.0.

**Conclusions:**

Distinguishing aerobic and muscle-strengthening adherence revealed patterns not evident from a single active–inactive classification. The cross-sectional design precludes conclusions about temporal order or causality.

## Highlights

• The pooled analysis included 22,344 adults with arthritis in the United States.
• Aerobic-only and combined adherence were associated with less mobility limitation.
• Combined versus neither showed 16.6 percentage points lower adjusted prevalence.
• Muscle-strengthening-only adherence showed a smaller association.
• Findings were similar across survey years and sensitivity analyses.

## 1 Introduction

Arthritis is a major source of functional limitation among U.S. adults. In 2023, approximately 24.8 million adults with doctor-diagnosed arthritis reported arthritis-attributable activity limitation, with a particularly high burden among those reporting difficulty walking or climbing steps (Stowe et al., 2026). Population-based evidence across osteoarthritis and rheumatoid arthritis likewise documents limitations in walking, stair climbing, daily activities, and social participation. Mobility limitation is therefore a central part of the functional burden of arthritis (Gikaro et al., 2022).

Exercise is a core component of arthritis management, and clinical guidelines consistently recommend aerobic and strengthening exercise tailored to individual capabilities and circumstances (Conley et al., 2023; England et al., 2023). These activities are separate components of federal physical activity guidelines, yet adherence to both remains low, particularly among older adults and people with poorer health (Garcia-Hermoso et al., 2023). A binary activity measure does not distinguish whether mobility limitation differs with aerobic adherence, muscle-strengthening adherence, or both.

Recent national studies among adults with arthritis have examined neighborhood correlates of walking or quantified arthritis-attributable activity limitation (Stowe et al., 2026; Pocha et al., 2025), but evidence relating mutually exclusive aerobic and muscle-strengthening guideline-adherence patterns to walking or stair-climbing difficulty specifically in this population remains limited. We therefore pooled 2020, 2022, and 2024 NHIS data to estimate relative and absolute associations for four leisure-time activity patterns. The prespecified primary comparison was both guidelines met versus neither guideline met; muscle-strengthening only versus neither, aerobic only versus neither, and both versus aerobic only were secondary comparisons. We also assessed the robustness of these associations through sensitivity analyses and examined potential heterogeneity across survey years and participant subgroups.

## 2 Methods

### 2.1 Study design and data source

This pooled cross-sectional study used the 2020, 2022, and 2024 National Health Interview Survey (NHIS) Sample Adult public-use files. NHIS is an annual, nationally representative household interview survey of the civilian, noninstitutionalized U.S. population that uses a geographically clustered probability sample; one adult aged 18 years or older is randomly selected within each sampled household for the Sample Adult interview (National Center for Health Statistics, n.d.). The leisure-time physical activity module was administered in 2020, 2022, and 2024 as part of the NHIS rotating core, but not in 2021 or 2023. The activity-guideline classification was available across the three selected cycles. The pooled model estimated a common association after adjustment for survey year rather than a temporal trend. Because the COVID-19 pandemic affected 2020 fieldwork, the primary analysis was repeated without that cycle.

### 2.2 Study population

Eligible respondents were Sample Adults aged 18 years or older who answered yes when asked whether a doctor or other health professional had ever told them that they had arthritis, rheumatoid arthritis, gout, lupus, or fibromyalgia. This broad NHIS case definition is referred to as doctor-diagnosed arthritis throughout the manuscript. Eligibility for the primary analysis required a valid four-category activity exposure, a valid primary mobility outcome, and complete data for all prespecified primary adjustment variables. The severe-mobility analysis used complete cases for the variables included in that model.

### 2.3 Leisure-time activity guideline adherence patterns

The exposure was the NHIS-derived four-category variable PA18_05R_A, which combines adherence to the federal aerobic and muscle-strengthening guidelines. The underlying NHIS questions assessed the frequency and duration of moderate- and vigorous-intensity leisure-time aerobic activity and the frequency of muscle-strengthening activity; year-specific source variables are documented in Supplementary Table S1. Aerobic adherence was defined as at least 150 min per week of moderate-intensity-equivalent leisure-time activity. Muscle-strengthening adherence was defined as muscle-strengthening activity performed at least twice per week (U.S. Department of Health and Human Services, 2018). Respondents were classified into four mutually exclusive patterns: neither guideline met, muscle-strengthening only, aerobic only, or both guidelines met. The neither-guideline group was the reference category. The measure represents leisure-time activity and does not capture occupational, transportation, or household activity.

### 2.4 Mobility limitation outcomes

Mobility limitation was derived from DIFF_A, which asks about difficulty walking or climbing steps. Responses indicated no difficulty, some difficulty, a lot of difficulty, or inability to perform the activity. The primary outcome, any mobility limitation, compared some difficulty, a lot of difficulty, or inability with no difficulty. Severe mobility limitation compared a lot of difficulty or inability with no or some difficulty. Refused, unknown, and not-ascertained responses were treated as missing.

### 2.5 Covariates

The primary adjustment set was selected before analysis using a prespecified causal framework and substantive knowledge rather than univariable associations or significance testing (Supplementary Fig. S1). Covariates were survey year; age, modeled with a natural cubic spline with four degrees of freedom; sex; race and ethnicity; educational attainment; family income-to-poverty category; marital status; U.S. census region; smoking status; body mass index (BMI) category; doctor-diagnosed diabetes; doctor-diagnosed hypertension; cardiovascular disease; chronic obstructive pulmonary disease; and cancer history. Cardiovascular disease was defined as any history of coronary heart disease, angina, myocardial infarction, or stroke. Depression, anxiety, and self-rated health were reserved for a prespecified extended-adjustment sensitivity analysis. Detailed definitions and harmonized coding are provided in Supplementary Table S1.

BMI category was included as a prespecified potential confounder because it may influence both activity participation and mobility. Because activity, BMI, and mobility were measured concurrently, BMI could also be a mediator or a consequence of mobility limitation; the model omitting BMI was therefore used to assess sensitivity to this uncertainty. The temporal roles of the included chronic conditions were also uncertain; an additional reduced-adjustment model therefore excluded BMI and these conditions.

### 2.6 Complex survey design and primary statistical analysis

Annual Sample Adult weights were divided by three so that pooled weighted totals represented the average annual population across the three cycles. Strata and primary sampling unit identifiers were made unique across survey years, and the complex survey design was defined before restriction to the arthritis domain.

Descriptive analyses reported unweighted sample sizes, survey-weighted means with standard errors, and survey-weighted proportions with 95% confidence intervals (CIs).

The primary association was estimated using survey-weighted modified Poisson regression with a log link and design-based standard errors. Results are reported as adjusted prevalence ratios (PRs) with 95% CIs. The prespecified primary comparison was both guidelines met versus neither guideline met and was tested at a two-sided alpha level of 0.05. Within the primary model, *P* values for the three secondary contrasts were adjusted using the Holm procedure. All other *P* values were two-sided and unadjusted.

Adjusted prevalences and prevalence differences were estimated by marginal standardization over the observed covariate distribution, with design-based 95% CIs. The modified Poisson model was the sole source of PRs. Because some fitted values from that model exceeded 1, a prespecified survey-weighted logistic companion model with the same covariates was used only to estimate marginal adjusted prevalences and absolute prevalence differences. No fitted values were truncated. Analyses were conducted in R version 4.6.1 (R Core Team, 2026) using the survey package version 4.5 (Lumley, 2004).

### 2.7 Secondary analyses, missing data, and preregistration

Severe mobility limitation was analyzed using the same modified Poisson approach. Prespecified supplementary analyses also examined the four-level ordinal mobility outcome and exploratory difficulties with errands and social participation. Heterogeneity across survey years was assessed using a joint Wald test for the interaction between activity pattern and survey year. Prespecified exploratory effect-modification analyses assessed sex, age group (18–64 years and ≥65 years), and obesity status; effect modification was evaluated using formal joint interaction tests. Sensitivity analyses excluded the 2020 cycle, omitted BMI from the adjustment set, added depression, anxiety, and self-rated health, or winsorized pooled weights at the 1st and 99th percentiles. Separate models were also fitted for each survey year. Additional sensitivity analyses fitted a reduced-adjustment model excluding BMI and the included chronic conditions and assessed the both-versus-aerobic-only contrast with and without adjustment for reconstructed weekly aerobic volume among participants with valid dose data.

Exposure and outcome values were not imputed. The primary analysis used complete cases for the exposure, outcome, and prespecified covariates. Among respondents with valid exposure and outcome data, 1055 of 23,399 (4.5%) were excluded because of missing covariates. As a sensitivity analysis, inverse-probability weights for complete-case inclusion were estimated among all arthritis-eligible respondents using survey year, age (linear and quadratic terms), sex, race and ethnicity, region, and income-to-poverty category. The weights were applied to the primary model, with uncertainty estimated using 400 stratified primary sampling unit bootstrap replicates that refitted both models.

The protocol and statistical analysis plan were registered on the Open Science Framework before the prespecified complex-survey analyses began (https://doi.org/10.17605/OSF.IO/DQ982). Public data access and variable-feasibility checks preceded registration.

Reporting followed the Strengthening the Reporting of Observational Studies in Epidemiology (STROBE) guidelines (Supplementary Checklist).

The NHIS protocols were reviewed and approved by the National Center for Health Statistics Research Ethics Review Board, and all participants provided informed consent in accordance with the annual survey procedures (National Center for Health Statistics, n.d.; National Center for Health Statistics, 2020; National Center for Health Statistics, 2022; National Center for Health Statistics, 2024). The present study used only deidentified public-use records and involved no participant contact or access to identifiable information; therefore, no additional participant consent was required for this secondary analysis.

## 3 Results

### 3.1 Study sample and participant characteristics

Of 24,319 adults meeting the study definition of doctor-diagnosed arthritis, 23,399 had valid activity-pattern and mobility data, and 22,344 had complete covariate data for the primary analysis. The final sample included 7644 respondents from 2020, 6675 from 2022, and 8025 from 2024 (Fig. 1; Supplementary Table S2).

**Fig. 1:**
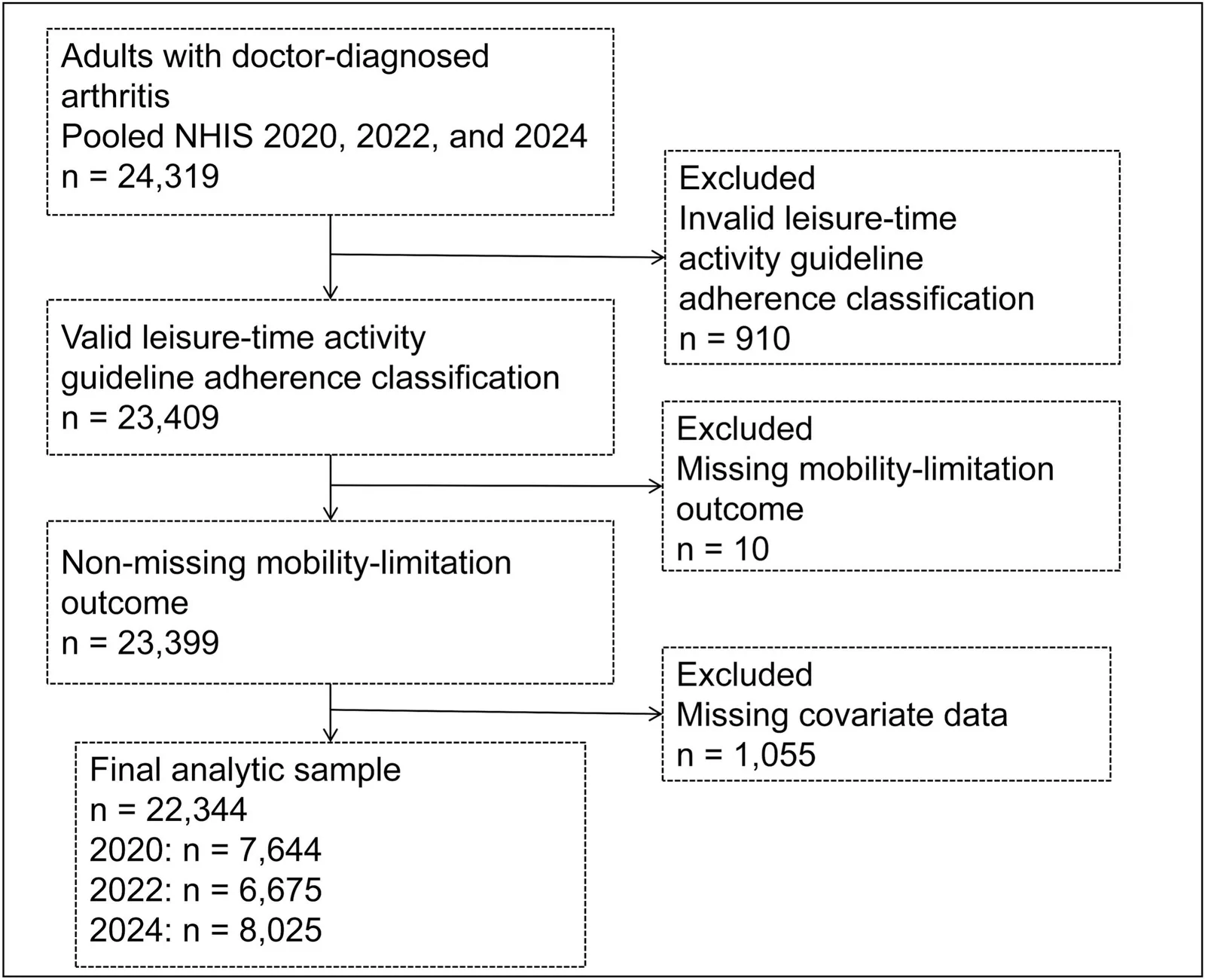
Participant flow for the pooled cross-sectional analysis of adults with doctor-diagnosed arthritis in the United States, 2020, 2022, and 2024. Note. The pooled Sample Adult survey files included 91,848 respondents; 24,319 met the study definition of doctor-diagnosed arthritis. Of these, 910 were excluded because of invalid activity-pattern data, 10 because of invalid mobility-outcome data, and 1055 because of missing prespecified covariates, yielding 22,344 complete cases for the primary analysis. Final analytical sample sizes were 7644 in 2020, 6675 in 2022, and 8025 in 2024. Exclusions are sequential.

Most participants met neither guideline (57.7%), whereas 14.5% met both guidelines; the remaining participants met the aerobic guideline only or the muscle-strengthening guideline only. Adults meeting both guidelines were younger on average than those meeting neither guideline (58.5 versus 64.2 years) and more often had a bachelor's degree or higher (46.4% versus 19.3%). Selected characteristics are presented in Table 1, with complete estimates in Supplementary Table S3.

**Table 1:** Selected characteristics of adults with doctor-diagnosed arthritis in the United States by leisure-time activity guideline adherence pattern, 2020, 2022, and 2024.

Characteristic	Neither guideline met	Muscle-strengthening only	Aerobic only	Both guidelines met
Sample size	12,663	1606	4764	3311
Activity-pattern distribution, %	57.7 (56.8, 58.6)	6.8 (6.4, 7.3)	21.0 (20.3, 21.6)	14.5 (13.9, 15.1)
Age, years	64.2 (0.2)	63.3 (0.5)	62.2 (0.3)	58.5 (0.4)
Female, %	62.1 (61.0, 63.2)	57.2 (54.3, 60.1)	54.2 (52.5, 56.0)	50.1 (48.1, 52.2)
Non-Hispanic White only, %	71.0 (69.7, 72.2)	73.9 (71.0, 76.8)	77.4 (75.7, 79.1)	78.8 (77.0, 80.7)
Bachelor's degree or higher, %	19.3 (18.5, 20.1)	29.8 (27.2, 32.5)	31.3 (29.7, 32.9)	46.4 (44.3, 48.4)
Married/cohabiting, %	58.7 (57.6, 59.7)	60.2 (57.5, 63.0)	65.4 (63.8, 67.0)	68.6 (66.8, 70.4)
Current smoker, %	16.1 (15.3, 17.0)	11.7 (9.7, 13.7)	13.7 (12.5, 15.0)	8.2 (6.9, 9.4)
Obesity, %	48.6 (47.5, 49.7)	41.5 (38.5, 44.4)	38.3 (36.6, 40.1)	30.2 (28.2, 32.2)
Doctor-diagnosed diabetes, %	24.0 (23.1, 25.0)	17.9 (15.6, 20.2)	14.5 (13.3, 15.8)	7.8 (6.7, 8.8)
Cardiovascular disease, %	23.5 (22.6, 24.4)	18.2 (16.0, 20.4)	16.1 (14.8, 17.3)	10.7 (9.5, 11.9)
Chronic obstructive pulmonary disease, %	15.2 (14.5, 16.0)	11.3 (9.4, 13.1)	9.3 (8.2, 10.3)	5.4 (4.6, 6.3)

Note. Values are unweighted sample sizes, survey-weighted means (standard errors), or survey-weighted percentages (95% confidence intervals), as indicated. Complete category distributions are reported in Supplementary Table S3.

### 3.2 Primary associations

The unadjusted survey-weighted prevalence of mobility limitation was 58.8% (95% CI, 57.7, 59.8) for neither guideline met, 49.0% (95% CI, 45.9, 52.1) for muscle-strengthening only, 35.3% (95% CI, 33.6, 37.1) for aerobic only, and 25.5% (95% CI, 23.8, 27.3) for both guidelines met.

After adjustment, the both-guidelines group had lower mobility-limitation prevalence than the neither-guideline group (PR, 0.62; 95% CI, 0.58, 0.66). Adjusted prevalences were 37.2% and 53.8%, respectively, corresponding to a difference of −16.6 percentage points (95% CI, −18.9, −14.3). The corresponding PRs were 0.72 for aerobic-only adherence and 0.94 for muscle-strengthening-only adherence; the adjusted prevalence difference between the both-guidelines and aerobic-only groups was −2.6 percentage points (Table 2; Fig. 2).

**Table 2:** Adjusted associations and marginal prevalence of any mobility limitation by leisure-time activity guideline adherence among adults with doctor-diagnosed arthritis in the United States, 2020, 2022, and 2024.

Activity pattern or contrast	Adjusted prevalence ratio (95% confidence interval)	Adjusted prevalence, % (95% confidence interval)	Adjusted prevalence difference, percentage points (95% confidence interval)
Neither guideline met	1.00	53.8 (52.7, 54.9)	Reference
Muscle-strengthening only	0.94 (0.88, 0.99)	50.3 (47.6, 53.1)	−3.4 (−6.3, −0.6)
Aerobic only	0.72 (0.69, 0.76)	39.9 (38.2, 41.5)	−13.9 (−15.9, −12.0)
Both guidelines met	0.62 (0.58, 0.66)	37.2 (35.2, 39.3)	−16.6 (−18.9, −14.3)
Both versus aerobic only	0.86 (0.79, 0.93)	–	−2.6 (−5.2, −0.1)

Note. Prevalence ratios were estimated using survey-weighted modified Poisson regression. Adjusted prevalences and prevalence differences were estimated using the survey-weighted logistic companion model. Category-specific prevalence differences use neither guideline met as the reference. Model sample size = 22,344.

**Fig. 2:**
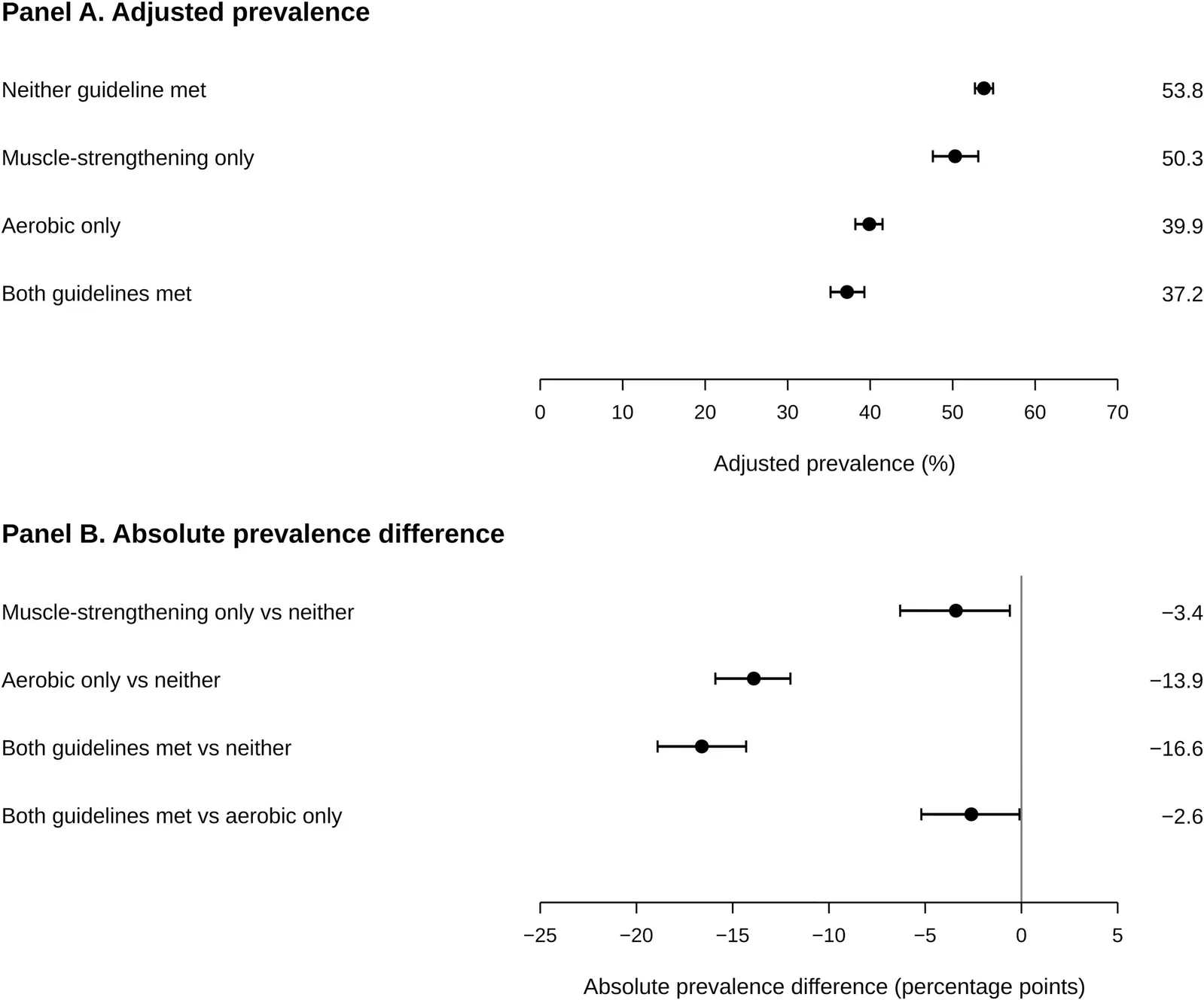
Adjusted marginal prevalence and absolute prevalence differences in any mobility limitation by leisure-time activity guideline adherence among adults with doctor-diagnosed arthritis in the United States, 2020, 2022, and 2024. Note. Panel A presents adjusted marginal prevalences for neither guideline met, muscle-strengthening only, aerobic only, and both guidelines met. Panel B presents the adjusted prevalence differences for muscle-strengthening only, aerobic only, and both guidelines met versus neither guideline met, and both guidelines met versus aerobic only. Error bars denote 95% confidence intervals. Adjusted marginal prevalences and prevalence differences were obtained from the survey-weighted logistic companion model using marginal standardization (*n* = 22,344).

### 3.3 Sensitivity, year-specific, and effect-modification analyses

The both-versus-neither estimate remained below 1.0 across prespecified sensitivity analyses, including exclusion of the 2020 cycle, omission of BMI, extended covariate adjustment, and weight winsorization (Supplementary Table S4). Associations were in the same direction across survey years, with no evidence of activity-pattern-by-year interaction (*P* = 0.273; Supplementary Table S5, Panel A).

There was no evidence of interaction by sex (joint *P* = 0.266) or age group (joint *P* = 0.145). The joint activity-pattern-by-obesity test yielded *P* < 0.001. For the primary contrast, the stratified PR was 0.59 (95% CI, 0.53, 0.65) among adults without obesity and 0.69 (95% CI, 0.62, 0.76) among adults with obesity. Interaction *P* values were unadjusted (Supplementary Table S5, Panel B).

The reduced-adjustment model yielded a both-versus-neither PR of 0.55 (95% CI, 0.51, 0.59). Median weekly aerobic-equivalent minutes were 315 in the aerobic-only group and 420 in the both-guidelines group; after adjustment for aerobic volume among 8024 respondents, the both-versus-aerobic-only PR was 0.92 (95% CI, 0.85, 1.00). Inverse-probability weighting for complete-case inclusion yielded a both-versus-neither PR of 0.62 (95% CI, 0.58, 0.66) (Supplementary Table S4).

### 3.4 Severe mobility limitation

Combined guideline adherence was also associated with a lower prevalence of severe mobility limitation compared with meeting neither guideline (PR, 0.41; 95% CI, 0.34, 0.49; Supplementary Table S5, Panel C). The prespecified ordinal model showed evidence against the proportional-odds assumption; exploratory functional-outcome estimates are presented in Supplementary Table S5.

## 4 Discussion

Meeting both guidelines was associated with lower mobility-limitation prevalence than meeting neither guideline, with differences on both relative and absolute scales. Aerobic-only adherence was also associated with lower prevalence, whereas the muscle-strengthening-only estimate was closer to 1.0. The primary estimate remained below 1.0 across sensitivity analyses and survey years, with no evidence of heterogeneity by year. Because activity and mobility were measured cross-sectionally, temporal order cannot be established, and reverse causation remains possible.

Previous population-based studies have generally combined physical activity into a single exposure, examined walking or guideline adherence as the outcome, or assessed functional measures other than mobility. Sung et al. reported an association between combined aerobic and resistance activity and handgrip strength in Korean adults but did not assess mobility or restrict the sample to adults with arthritis (Sung et al., 2022). Walter et al. examined aerobic and muscle-strengthening guideline adherence in Native Hawaiian and Pacific Islander adults and found fewer functional limitations among those meeting both guidelines than among those meeting neither, although results varied across comparisons (Walter et al., 2022). Son et al. found that aerobic-only, muscle-strengthening-only, and combined adherence were each associated with higher odds of no overall functional limitation in older Korean adults (Son, 2026). In a four-year Osteoarthritis Initiative analysis, Kiehl et al. reported less mobility disability among participants with lifelong strength-training participation than among those with no lifetime participation (Kiehl et al., 2026). These studies differ in populations, functional outcomes, and effect measures, precluding direct comparisons of association magnitudes. Recent NHIS studies have examined neighborhood correlates of walking and arthritis-attributable activity limitation (Stowe et al., 2026; Pocha et al., 2025). Our analysis separated aerobic and muscle-strengthening adherence among adults with doctor-diagnosed arthritis and estimated both relative and absolute associations with difficulty walking or climbing steps.

Trial syntheses in knee and hip osteoarthritis support functional benefits from aerobic and resistance exercise (Yan et al., 2025; Marriott et al., 2024), but they do not establish the direction of the present cross-sectional association. The smaller muscle-strengthening-only association may reflect limited measurement of strengthening dose and selective participation by adults unable to meet aerobic targets; the both-versus-aerobic-only contrast cannot isolate muscle-strengthening adherence from differences in aerobic volume. These findings neither demonstrate a biological interaction between activity components nor show that muscle-strengthening exercise is ineffective.

Reverse causation is a central alternative explanation: adults with less pain, fatigue, or mobility difficulty may be more able to meet aerobic and strengthening guidelines. Arthritis severity, treatment access, neighborhood conditions, social resources, and unmeasured physical capacity may influence both guideline adherence and mobility. Although the models adjusted for demographic, socioeconomic, behavioral, and clinical factors, residual confounding remains possible. The stronger association in the reduced-adjustment model indicates sensitivity to covariate selection but does not establish mediation.

The exploratory obesity interaction should be interpreted cautiously because estimates remained below 1.0 in both strata, interaction testing was not adjusted for multiple comparisons, and the finding requires external replication. It should not be used to support different exercise recommendations.

The analysis drew on a large sample from nationally representative NHIS data, harmonized three survey cycles, and preserved the complex survey design. Covariates were selected before analysis using a prespecified causal framework. Reporting PRs, adjusted prevalences, and absolute differences allowed the associations to be viewed on both relative and absolute scales. The primary result was also examined across prespecified sensitivity analyses.

Several limitations warrant emphasis. Arthritis, activity, and mobility were self-reported, and the cross-sectional design precludes temporal ordering. The NHIS arthritis item identifies an ever-diagnosed group and combines conditions with different symptom profiles and functional consequences; it did not distinguish subtypes, duration, anatomical site, current disease activity, or severity. Condition-specific associations may therefore have been obscured. The exposure captured leisure-time activity only, excluding occupational, household, and transportation activity, and did not characterize specific exercise modes or training features. The both-versus-aerobic-only contrast also differed in aerobic volume, limiting attribution to muscle-strengthening adherence. Harmonized measures of pain and arthritis severity were unavailable, and mobility was assessed by reported walking or stair-climbing difficulty rather than performance-based testing.

The findings may not generalize to institutionalized adults, who are outside the NHIS target population. Complete-case analysis could introduce selection bias if missingness was related to both activity and mobility after accounting for measured covariates, although inverse-probability-weighted estimates were similar. Conditions specific to 2020 may also have influenced activity reporting, although excluding that year did not materially alter the primary estimate. The primary estimate was similar when BMI was omitted, but the temporal roles of BMI and other chronic conditions remain uncertain, leaving potential overadjustment unresolved.

## 5 Conclusions

Among adults with doctor-diagnosed arthritis, aerobic-only and combined guideline adherence were associated with lower mobility-limitation prevalence, while the association was smaller for muscle-strengthening-only adherence. These cross-sectional associations do not establish temporal direction. Longitudinal studies with repeated activity assessments and objective measures of physical function are needed.

## Declaration of generative AI and AI-assisted technologies in the manuscript preparation process

During the preparation of this work, the authors used ChatGPT™ (OpenAI) to assist with manuscript organization, language editing, and technical formatting. After using this tool, the authors reviewed and edited the content as needed and take full responsibility for the content of the publication.

## CRediT authorship contribution statement

**Dejin Wang:** Writing – review & editing. **Jueliang Tian:** Investigation. **Yuxi Peng:** Project administration, Data curation. **Shaoxue Wu:** Validation, Methodology. **Yuhuan Feng:** Writing – review & editing, Conceptualization.

## Code availability

The R code used for data harmonization, complex-survey analysis, and generation of the reported results is available through the Open Science Framework repository at https://doi.org/10.17605/OSF.IO/DQ982.

## Funding

This research received no specific grant from any funding agency in the public, commercial, or not-for-profit sectors.

## Declaration of competing interest

The authors declare that they have no known competing financial interests or personal relationships that could have appeared to influence the work reported in this paper.

## Figures and Tables

**Supplementary Figure S1:**
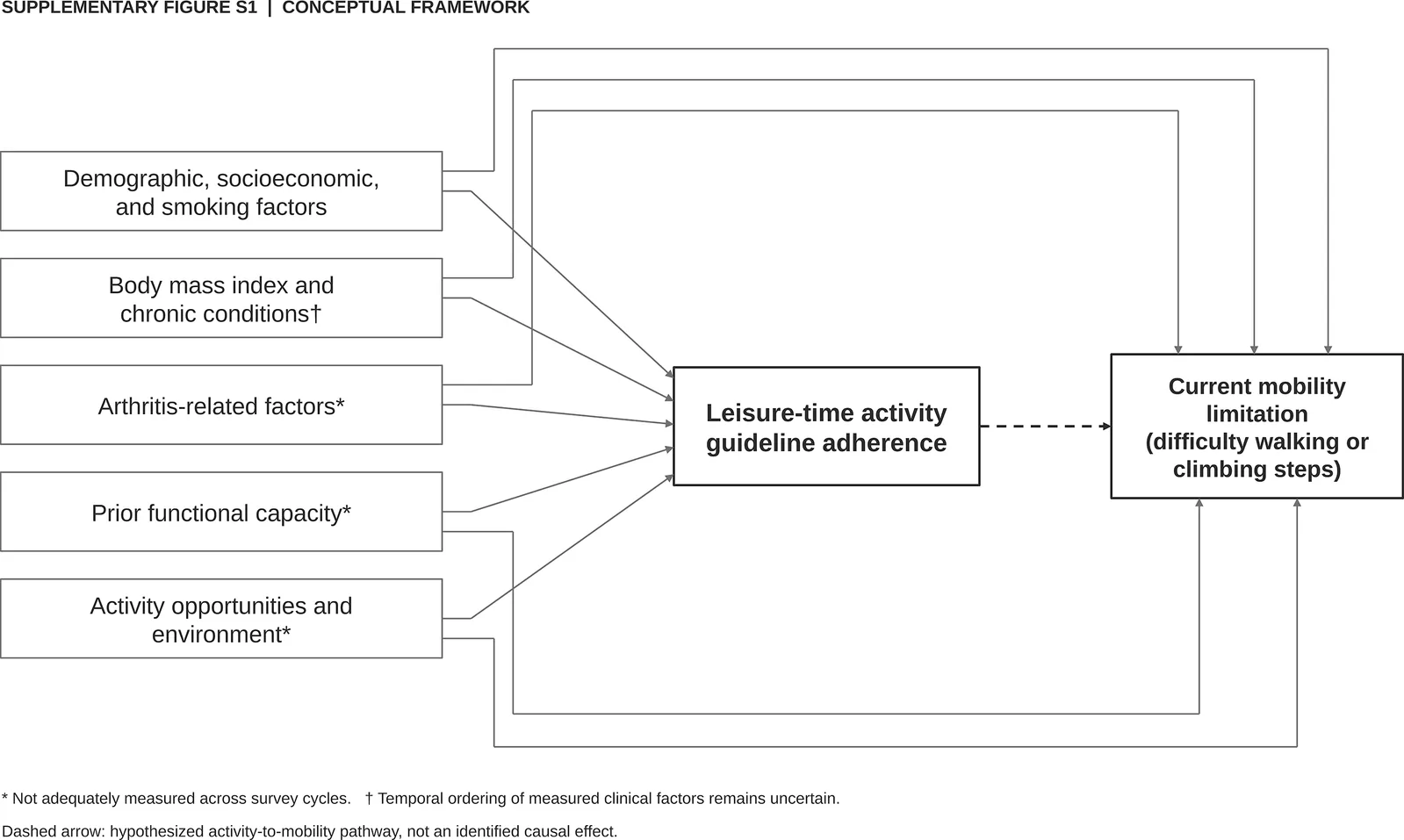
Supporting information and a conceptual framework for the analysis of leisure-time aerobic and muscle-strengthening guideline adherence and mobility limitation among adults with doctor-diagnosed arthritis in the United States, using pooled NHIS data from 2020, 2022, and 2024. Note. The diagram summarizes the conceptual factors considered in selecting the adjustment set and interpreting the cross-sectional associations. Measured demographic, socioeconomic, and behavioral factors included survey year, age, sex, race and ethnicity, educational attainment, income-to-poverty category, marital status, census region, and smoking status. Measured clinical factors included body mass index, diabetes, hypertension, cardiovascular disease, chronic obstructive pulmonary disease, and cancer history. Arthritis severity, pain, disease history, prior functional capacity, and activity opportunities were not adequately measured across survey cycles. The proposed activity-to-mobility pathway represents a conceptual hypothesis rather than an identified causal effect. Prior functional capacity is represented as a possible antecedent of both current activity and mobility limitation. The temporal roles of body mass index and chronic conditions remain uncertain because exposure, outcome, and covariates were measured concurrently. These uncertainties motivated the reduced-adjustment sensitivity analysis. Arrows represent conceptual assumptions, not causal effects established by the data.

## Data Availability

The 2020, 2022, and 2024 NHIS Sample Adult public-use data and documentation are available from the National Center for Health Statistics (https://www.cdc.gov/nchs/nhis/documentation/index.html). The study protocol, statistical analysis plan, harmonization documentation, and aggregate data underlying the reported results are available through the Open Science Framework repository at https://doi.org/10.17605/OSF.IO/DQ982.

## References

[bb0005] Conley B. (2023). Core recommendations for osteoarthritis care: a systematic review of clinical practice guidelines. Arthritis Care Res..

[bb0010] England B.R. (2023). 2022 American College of Rheumatology guideline for exercise, rehabilitation, diet, and additional integrative interventions for rheumatoid arthritis. Arthritis Rheum..

[bb0015] Garcia-Hermoso A. (2023). Adherence to aerobic and muscle-strengthening activities guidelines: a systematic review and meta-analysis of 3.3 million participants across 32 countries. Br. J. Sports Med..

[bb0020] Gikaro J.M., Xiong H., Lin F. (2022). Activity limitation and participation restriction in osteoarthritis and rheumatoid arthritis: findings based on the National Health and Nutritional Examination Survey. BMC Musculoskelet. Disord..

[bb0025] Kiehl D., Thompson Z., Johnson A.J., Sibille K.T., Vincent K.R., Vincent H.K. (2026). Relationship of strength training lifetime exposure with functional outcomes and mobility over 4 years: data from the Osteoarthritis Initiative. J. Sport Health Sci..

[bb0030] Lumley T. (2004). Analysis of complex survey samples. J. Stat. Softw..

[bb0035] Marriott K.A. (2024). Are the effects of resistance exercise on pain and function in knee and hip osteoarthritis dependent on exercise volume, duration, and adherence? A systematic review and meta-analysis. Arthritis Care Res..

[bb0040] National Center for Health Statistics (2020). https://www.cdc.gov/nchs/nhis/documentation/2020-nhis.html.

[bb0045] National Center for Health Statistics (2022). https://www.cdc.gov/nchs/nhis/documentation/2022-nhis.html.

[bb0050] National Center for Health Statistics (2024). https://www.cdc.gov/nchs/nhis/documentation/2024-nhis.html.

[bb0055] National Center for Health Statistics. NHIS methods. Centers for Disease Control and Prevention. https://www.cdc.gov/nchs/nhis/about/nhis-methods.html (accessed July 27, 2026).

[bb0060] Pocha C.C. (2025). Neighborhood characteristics and walking behavior among adults with arthritis: a National Health Interview Survey study. Arthritis Care Res..

[bb0065] R Core Team (2026). https://www.R-project.org/.

[bb0070] Son N. (2026). Association between physical activity levels and life functioning in Korean older adults: a cross-sectional analysis of the 2024 Korea National Health and Nutrition Examination Survey. Healthcare (Basel).

[bb0075] Stowe E.W. (2026). Prevalence of arthritis-attributable activity limitations—United States, 2023. Arthritis Care Res..

[bb0080] Sung J.H., Son S.R., Baek S.H., Kim B.J. (2022). The association of aerobic, resistance, and combined exercises with the handgrip strength of middle-aged and elderly Korean adults: a nationwide cross-sectional study. BMC Geriatr..

[bb0085] U.S. Department of Health and Human Services (2018).

[bb0090] Walter C.S., Narcisse M.R., Vincenzo J.L., Felix H.C., McElfish P.A. (2022). Associations between physical activity and functional limitations in native Hawaiian and Pacific Islander middle-aged and older adults in the United States. Ethn. Health.

[bb0095] Yan L. (2025). Comparative efficacy and safety of exercise modalities in knee osteoarthritis: systematic review and network meta-analysis. BMJ.

