# Supplementary material for: Aerobic and muscle-strengthening guideline adherence and mobility limitation among adults with arthritis in the United States: a pooled cross-sectional analysis, 2020–2024
Source: Prev Med Rep. 2026 Oct 29;71:103692. doi: 10.1016/j.pmedr.2026.103692 (PMC13744215; doi:10.1016/j.pmedr.2026.103692)
Supplement: Supplementary file 1 [file mmc2.docx]

**Supplementary Table S1. Variable definitions, survey-year-specific activity source variables, and harmonized analytic coding among adults with doctor-diagnosed arthritis in the United States, 2020, 2022, and 2024.**

| **Construct** | **Survey variable(s)** | **Years** | **Analytic coding** | **Role** |
| --- | --- | --- | --- | --- |
| Survey year | Derived from source file | 2020, 2022, 2024 | Categorical fixed effect | Design covariate |
| Doctor-diagnosed arthritis | ARTHEV_A | 2020, 2022, 2024 | Yes vs no; broad NHIS arthritis item | Domain population |
| Activity guideline-adherence pattern | PA18_05R_A | 2020, 2022, 2024 | Neither guideline met; muscle-strengthening only; aerobic only; both guidelines met. Aerobic adherence: at least 150 moderate-intensity-equivalent minutes per week; muscle-strengthening adherence: at least twice weekly | Primary exposure |
| Leisure-time activity source items | MODN_A, MODTP_A, MODLN_A, MODLTP_A, VIGN_A, VIGTP_A, VIGLN_A, VIGLTP_A, STRN_A, STRTP_A | 2020, 2022 | Questionnaire source variables underlying the official activity classification | Exposure source documentation |
| Leisure-time activity source items | MODNR_A, MODTPR_A, MODLNR_A, MODLTPR_A, VIGNR_A, VIGTPR_A, VIGLNR_A, VIGLTPR_A, STRNR_A, STRTPR_A | 2024 | Year-specific questionnaire source variables underlying the official activity classification | Exposure source documentation |
| Any mobility limitation | DIFF_A | 2020, 2022, 2024 | Some/a lot of difficulty or unable vs no difficulty | Primary outcome |
| Severe mobility limitation | DIFF_A | 2020, 2022, 2024 | A lot of difficulty or unable vs no/some difficulty | Secondary outcome |
| Age | AGEP_A | 2020, 2022, 2024 | Continuous, 18–85 years (85 top-coded); natural cubic spline with four degrees of freedom, interior knots at 58, 68, and 76 years, and boundary knots at 18 and 85 years | Primary covariate |
| Sex | SEX_A | 2020, 2022, 2024 | Male; female | Primary covariate / modifier |
| Race and ethnicity | HISPALLP_A | 2020, 2022, 2024 | Seven public-use categories | Primary covariate |
| Educational attainment | EDUC_A (2020); EDUCP_A (2022, 2024) | 2020, 2022, 2024 | Less than high school; high school or General Educational Development credential; some college or associate degree; bachelor’s degree or higher | Primary covariate |
| Family income-to-poverty category | RATCAT_A | 2020, 2022, 2024 | Fourteen public-use categories | Primary covariate |
| Marital status | MARSTAT_A | 2020, 2022, 2024 | Married/cohabiting; formerly married; never married | Primary covariate |
| United States census region | REGION | 2020, 2022, 2024 | Northeast; Midwest; South; West | Primary covariate |
| Smoking status | SMKCIGST_A | 2020, 2022, 2024 | Never; former; current smoker | Primary covariate |
| Body mass index category | BMICAT_A | 2020, 2022, 2024 | Underweight; healthy weight; overweight; obese | Primary covariate / modifier |
| Diabetes | DIBEV_A | 2020, 2022, 2024 | Doctor-diagnosed history: yes vs no | Primary covariate |
| Hypertension | HYPEV_A | 2020, 2022, 2024 | Doctor-diagnosed history: yes vs no | Primary covariate |
| Cardiovascular disease | CHDEV_A; ANGEV_A; MIEV_A; STREV_A | 2020, 2022, 2024 | Any coronary heart disease, angina, myocardial infarction, or stroke | Primary covariate |
| Chronic obstructive pulmonary disease | COPDEV_A | 2020, 2022, 2024 | Doctor-diagnosed history: yes vs no | Primary covariate |
| Cancer history | CANEV_A | 2020, 2022, 2024 | Any cancer history: yes vs no | Primary covariate |
| Depression history | DEPEV_A | 2020, 2022, 2024 | Yes vs no | Sensitivity covariate |
| Anxiety history | ANXEV_A | 2020, 2022, 2024 | Yes vs no | Sensitivity covariate |
| Self-rated health | PHSTAT_A | 2020, 2022, 2024 | Excellent; very good; good; fair; poor | Sensitivity covariate |
| Survey design variables | WTFA_A; PSTRAT; PPSU | 2020, 2022, 2024 | Annual sample-adult weights divided by three; strata and primary sampling unit identifiers made unique across survey years | Survey design |

Note. Refused, not ascertained, unknown, and invalid responses were treated as missing according to the relevant survey-year codebooks. The official four-category leisure-time activity guideline-adherence variable (PA18_05R_A) was retained across all three survey cycles. The source-variable mapping documents differences in questionnaire variable names between 2020/2022 and 2024 and does not establish identical questionnaire wording or recoding procedures.

**Supplementary Table S2. Participant selection, characteristics of included and excluded respondents, and covariate missingness among adults with doctor-diagnosed arthritis in the United States, 2020, 2022, and 2024.**

**Panel A. Sample selection and exclusions**

| **Item** | **Survey year** | **Previous sample size** | **Remaining sample size** | **Excluded respondents** |
| --- | --- | --- | --- | --- |
| All pooled Sample Adults | 2020 | 31,568 | 31,568 | 0 |
| All pooled Sample Adults | 2022 | 27,651 | 27,651 | 0 |
| All pooled Sample Adults | 2024 | 32,629 | 32,629 | 0 |
| All pooled Sample Adults | Pooled | 91,848 | 91,848 | 0 |
| Doctor-diagnosed arthritis | 2020 | 31,568 | 8,322 | 23,246 |
| Doctor-diagnosed arthritis | 2022 | 27,651 | 7,283 | 20,368 |
| Doctor-diagnosed arthritis | 2024 | 32,629 | 8,714 | 23,915 |
| Doctor-diagnosed arthritis | Pooled | 91,848 | 24,319 | 67,529 |
| Valid four-category activity pattern | 2020 | 8,322 | 8,024 | 298 |
| Valid four-category activity pattern | 2022 | 7,283 | 7,002 | 281 |
| Valid four-category activity pattern | 2024 | 8,714 | 8,383 | 331 |
| Valid four-category activity pattern | Pooled | 24,319 | 23,409 | 910 |
| Valid primary mobility outcome after valid exposure | 2020 | 8,024 | 8,020 | 4 |
| Valid primary mobility outcome after valid exposure | 2022 | 7,002 | 7,001 | 1 |
| Valid primary mobility outcome after valid exposure | 2024 | 8,383 | 8,378 | 5 |
| Valid primary mobility outcome after valid exposure | Pooled | 23,409 | 23,399 | 10 |
| Primary covariates complete | 2020 | 8,020 | 7,644 | 376 |
| Primary covariates complete | 2022 | 7,001 | 6,675 | 326 |
| Primary covariates complete | 2024 | 8,378 | 8,025 | 353 |
| Primary covariates complete | Pooled | 23,399 | 22,344 | 1,055 |

**Panel B. Characteristics of included and excluded respondents**

| **Characteristic** | **Included complete cases (n=22,344)** | **Excluded: invalid exposure or outcome (n=920)** | **Excluded: missing covariates (n=1,055)** |
| --- | --- | --- | --- |
| Age, years, weighted mean | 62.9 | 63.7 | 63.9 |
| Female, % | 58.4 | 55.9 | 71.8 |
| Non-Hispanic White, % | 73.6 | 69.1 | 68.3 |
| Bachelor's degree or higher, % | 26.5 | 17.5 | 21.0 |
| Current smoker, % | 14.2 | 22.2 | 13.5 |
| Any mobility limitation, % | 48.4 | 51.0 | 60.4 |

**Panel C. Covariate-specific missingness**

| **Covariate** | **Respondents with missing data** |
| --- | --- |
| Body mass index | 507 |
| Marital status | 277 |
| Educational attainment | 116 |
| Cardiovascular disease | 104 |
| Smoking status | 34 |

**Panel C (continued). Covariate-specific missingness**

| **Covariate** | **Respondents with missing data** |
| --- | --- |
| Chronic obstructive pulmonary disease | 22 |
| Hypertension | 20 |
| Cancer history | 14 |
| Diabetes | 11 |
| Sex | 1 |

Note. Panel A presents unweighted sample counts at each stage of participant selection. Panel B presents survey-weighted means and percentages. Respondents excluded because of invalid activity classification or mobility outcome data (n = 920) are distinguished from those excluded because of missing primary covariates (n = 1,055). Descriptive estimates are based on available responses for each characteristic; the valid denominator may differ across variables. Panel C reports covariate-specific missing counts among 23,399 respondents with valid exposure and outcome data. Because individual respondents could have missing values for more than one covariate, the sum of covariate-specific missing counts exceeds the number of respondents excluded from the complete-case analysis. Valid denominators for age, female sex, and non-Hispanic White were 22,344, 920, and 1,055, respectively, across the included complete-case, invalid-exposure-or-outcome, and missing-covariate groups. For bachelor’s degree or higher, the corresponding valid denominators were 22,344, 907, and 939; for current smoking, 22,344, 447, and 1,021; and for any mobility limitation, 22,344, 908, and 1,055.

**Supplementary Table S3. Detailed characteristics of adults with doctor-diagnosed arthritis in the United States by leisure-time activity guideline adherence pattern, 2020, 2022, and 2024.**

| **Characteristic** | **Level or statistic** | **Neither guideline met** | **Muscle-strengthening only** | **Aerobic only** | **Both guidelines met** |
| --- | --- | --- | --- | --- | --- |
| Sample size | Unweighted sample size | 12,663 | 1,606 | 4,764 | 3,311 |
| Activity-pattern distribution | Survey-weighted percentage (95% confidence interval) | 57.7 (56.8, 58.6)% | 6.8 (6.4, 7.3)% | 21.0 (20.3, 21.6)% | 14.5 (13.9, 15.1)% |
| Age, years | Survey-weighted mean (standard error) | 64.2 (0.2) | 63.3 (0.5) | 62.2 (0.3) | 58.5 (0.4) |
| Sex | Male | 37.9 (36.8, 39.0)% | 42.8 (39.9, 45.7)% | 45.8 (44.0, 47.5)% | 49.9 (47.8, 51.9)% |
| Sex | Female | 62.1 (61.0, 63.2)% | 57.2 (54.3, 60.1)% | 54.2 (52.5, 56.0)% | 50.1 (48.1, 52.2)% |
| Race and ethnicity | Hispanic | 10.9 (10.0, 11.9)% | 9.5 (7.5, 11.5)% | 8.0 (6.9, 9.1)% | 7.1 (6.0, 8.3)% |
| Race and ethnicity | Non-Hispanic White only | 71.0 (69.7, 72.2)% | 73.9 (71.0, 76.8)% | 77.4 (75.7, 79.1)% | 78.8 (77.0, 80.7)% |
| Race and ethnicity | Non-Hispanic Black/African American only | 12.4 (11.5, 13.3)% | 12.8 (10.5, 15.1)% | 8.5 (7.4, 9.6)% | 8.3 (7.0, 9.6)% |
| Race and ethnicity | Non-Hispanic Asian only | 3.2 (2.8, 3.7)% | 1.8 (1.1, 2.6)% | 3.7 (3.0, 4.4)% | 2.9 (2.2, 3.6)% |
| Race and ethnicity | Non-Hispanic American Indian or Alaska Native only | 0.8 (0.5, 1.1)% | 1.0 (0.4, 1.6)% | 0.6 (0.4, 0.9)% | 0.6 (0.2, 1.0)% |
| Race and ethnicity | Non-Hispanic American Indian or Alaska Native and any other group | 1.0 (0.8, 1.2)% | 0.6 (0.2, 1.0)% | 1.0 (0.7, 1.4)% | 0.9 (0.5, 1.4)% |
| Race and ethnicity | Other single and multiple races | 0.7 (0.5, 0.8)% | 0.3 (0.1, 0.6)% | 0.8 (0.4, 1.1)% | 1.2 (0.7, 1.8)% |
| Educational attainment | Less than high school | 15.8 (14.9, 16.7)% | 7.4 (5.7, 9.2)% | 10.1 (8.8, 11.4)% | 4.9 (3.9, 5.9)% |
| Educational attainment | High school or General Educational Development credential | 33.0 (32.0, 34.0)% | 27.3 (24.5, 30.1)% | 27.1 (25.4, 28.8)% | 17.6 (15.9, 19.3)% |
| Educational attainment | Some college or associate degree | 31.9 (30.9, 32.9)% | 35.5 (32.7, 38.3)% | 31.5 (29.9, 33.1)% | 31.2 (29.3, 33.1)% |
| Educational attainment | Bachelor's degree or higher | 19.3 (18.5, 20.1)% | 29.8 (27.2, 32.5)% | 31.3 (29.7, 32.9)% | 46.4 (44.3, 48.4)% |
| Family income-to-poverty category | 0.00-0.49 | 2.9 (2.5, 3.3)% | 1.3 (0.7, 1.9)% | 1.7 (1.3, 2.2)% | 1.1 (0.6, 1.5)% |
| Family income-to-poverty category | 0.50–0.74 | 4.6 (4.1, 5.1)% | 2.6 (1.5, 3.6)% | 2.4 (1.9, 2.9)% | 1.7 (1.1, 2.2)% |
| Family income-to-poverty category | 0.75–0.99 | 6.6 (6.1, 7.1)% | 4.8 (3.5, 6.2)% | 4.0 (3.3, 4.7)% | 2.7 (2.0, 3.5)% |
| Family income-to-poverty category | 1.00–1.24 | 5.6 (5.1, 6.0)% | 3.7 (2.6, 4.8)% | 4.5 (3.8, 5.3)% | 2.7 (2.0, 3.4)% |
| Family income-to-poverty category | 1.25–1.49 | 6.9 (6.3, 7.4)% | 4.5 (3.3, 5.7)% | 4.8 (3.8, 5.7)% | 2.3 (1.6, 3.0)% |
| Family income-to-poverty category | 1.50–1.74 | 5.4 (4.9, 5.9)% | 5.5 (4.1, 7.0)% | 4.8 (3.9, 5.7)% | 2.3 (1.7, 2.9)% |
| Family income-to-poverty category | 1.75–1.99 | 5.5 (5.1, 6.0)% | 4.5 (3.3, 5.7)% | 4.6 (3.9, 5.3)% | 3.5 (2.6, 4.3)% |
| Family income-to-poverty category | 2.00–2.49 | 10.7 (10.0, 11.3)% | 8.4 (6.7, 10.0)% | 9.1 (8.1, 10.1)% | 6.5 (5.5, 7.6)% |
| Family income-to-poverty category | 2.50–2.99 | 9.1 (8.5, 9.7)% | 8.4 (6.7, 10.0)% | 7.8 (6.8, 8.8)% | 5.9 (4.9, 7.0)% |
| Family income-to-poverty category | 3.00–3.49 | 7.2 (6.7, 7.8)% | 6.2 (4.9, 7.6)% | 6.3 (5.5, 7.1)% | 6.2 (5.3, 7.2)% |
| Family income-to-poverty category | 3.50–3.99 | 6.2 (5.7, 6.7)% | 7.6 (5.9, 9.3)% | 7.1 (6.1, 8.0)% | 6.6 (5.5, 7.7)% |
| Family income-to-poverty category | 4.00–4.49 | 5.0 (4.6, 5.5)% | 5.7 (4.5, 7.0)% | 5.7 (5.0, 6.5)% | 5.6 (4.7, 6.6)% |
| Family income-to-poverty category | 4.50–4.99 | 4.8 (4.3, 5.2)% | 6.2 (4.8, 7.6)% | 5.8 (5.0, 6.6)% | 6.9 (5.9, 8.0)% |
| Family income-to-poverty category | 5.00 or greater | 19.6 (18.8, 20.5)% | 30.6 (27.7, 33.4)% | 31.4 (29.7, 33.1)% | 45.9 (43.8, 48.1)% |
| Marital status | Married/cohabiting | 58.7 (57.6, 59.7)% | 60.2 (57.5, 63.0)% | 65.4 (63.8, 67.0)% | 68.6 (66.8, 70.4)% |
| Marital status | Formerly married | 31.8 (30.9, 32.7)% | 30.0 (27.6, 32.5)% | 26.1 (24.7, 27.4)% | 21.0 (19.6, 22.5)% |
| Marital status | Never married | 9.5 (8.9, 10.2)% | 9.7 (7.9, 11.6)% | 8.6 (7.5, 9.6)% | 10.4 (9.1, 11.7)% |
| United States census region | Northeast | 16.5 (15.6, 17.5)% | 16.5 (13.5, 19.4)% | 17.0 (15.6, 18.4)% | 17.5 (15.7, 19.3)% |
| United States census region | Midwest | 22.1 (21.0, 23.2)% | 23.7 (21.1, 26.3)% | 23.8 (22.2, 25.3)% | 21.1 (19.3, 22.8)% |
| United States census region | South | 42.0 (40.6, 43.4)% | 38.7 (35.4, 41.9)% | 35.9 (34.1, 37.7)% | 34.2 (31.9, 36.4)% |
| United States census region | West | 19.4 (18.2, 20.5)% | 21.1 (18.7, 23.6)% | 23.3 (21.7, 25.0)% | 27.3 (25.2, 29.4)% |
| Smoking status | Never smoker | 50.3 (49.1, 51.4)% | 53.4 (50.4, 56.4)% | 51.1 (49.3, 52.8)% | 57.7 (55.7, 59.8)% |
| Smoking status | Former smoker | 33.6 (32.6, 34.7)% | 34.9 (32.1, 37.7)% | 35.2 (33.6, 36.9)% | 34.1 (32.2, 36.0)% |
| Smoking status | Current smoker | 16.1 (15.3, 17.0)% | 11.7 (9.7, 13.7)% | 13.7 (12.5, 15.0)% | 8.2 (6.9, 9.4)% |
| Body mass index category | Underweight | 1.4 (1.2, 1.7)% | 0.9 (0.4, 1.4)% | 0.9 (0.6, 1.2)% | 1.4 (0.9, 1.9)% |
| Body mass index category | Healthy weight | 20.0 (19.2, 20.9)% | 21.2 (18.8, 23.6)% | 25.7 (24.2, 27.2)% | 31.3 (29.4, 33.2)% |
| Body mass index category | Overweight | 29.9 (28.9, 30.9)% | 36.4 (33.6, 39.3)% | 35.0 (33.4, 36.7)% | 37.1 (35.1, 39.1)% |
| Body mass index category | Obese | 48.6 (47.5, 49.7)% | 41.5 (38.5, 44.4)% | 38.3 (36.6, 40.1)% | 30.2 (28.2, 32.2)% |

**Supplementary Table S3 (continued)**

| **Characteristic** | **Level or statistic** | **Neither guideline met** | **Muscle-strengthening only** | **Aerobic only** | **Both guidelines met** |
| --- | --- | --- | --- | --- | --- |
| Doctor-diagnosed diabetes | Yes | 24.0 (23.1, 25.0)% | 17.9 (15.6, 20.2)% | 14.5 (13.3, 15.8)% | 7.8 (6.7, 8.8)% |
| Doctor-diagnosed hypertension | Yes | 63.6 (62.5, 64.6)% | 59.4 (56.4, 62.4)% | 53.1 (51.4, 54.9)% | 42.3 (40.2, 44.3)% |
| Cardiovascular disease composite | Yes | 23.5 (22.6, 24.4)% | 18.2 (16.0, 20.4)% | 16.1 (14.8, 17.3)% | 10.7 (9.5, 11.9)% |
| Chronic obstructive pulmonary disease | Yes | 15.2 (14.5, 16.0)% | 11.3 (9.4, 13.1)% | 9.3 (8.2, 10.3)% | 5.4 (4.6, 6.3)% |
| Cancer history | Yes | 20.4 (19.6, 21.3)% | 23.0 (20.6, 25.4)% | 19.7 (18.4, 21.1)% | 18.1 (16.6, 19.5)% |

Note. Values are unweighted sample sizes, survey-weighted means (standard errors), or survey-weighted percentages (95% confidence intervals), as indicated.

**Supplementary Table S4. Sensitivity analyses of associations between leisure-time activity guideline adherence and mobility limitation among adults with doctor-diagnosed arthritis in the United States, 2020, 2022, and 2024.**

| **Sensitivity analysis** | **Effect measure** | **Both guidelines vs neither (95% CI)** | **Both guidelines vs aerobic only (95% CI)** | **Model sample size** |
| --- | --- | --- | --- | --- |
| Excluding 2020 (2022 and 2024 only) | Prevalence ratio | 0.60 (0.55, 0.65) | 0.83 (0.75, 0.92) | 14,700 |
| Primary adjustment set without body mass index | Prevalence ratio | 0.58 (0.54, 0.63) | 0.83 (0.77, 0.90) | 22,819 |
| Primary adjustment plus depression, anxiety, and self-rated health | Prevalence ratio | 0.75 (0.70, 0.80) | 0.92 (0.85, 0.99) | 22,306 |
| Weights winsorized at the 1st and 99th percentiles | Prevalence ratio | 0.62 (0.57, 0.66) | 0.86 (0.79, 0.93) | 22,344 |

**Supplementary Table S4 (continued)**

| **Sensitivity analysis** | **Effect measure** | **Both guidelines vs neither (95% CI)** | **Both guidelines vs aerobic only (95% CI)** | **Model sample size** |
| --- | --- | --- | --- | --- |
| Reduced-adjustment model excluding body mass index and chronic conditions | Prevalence ratio | 0.55 (0.51, 0.59) | 0.82 (0.75, 0.89) | 22,344 |
| Aerobic-adherent subgroup, without additional aerobic-volume adjustment | Prevalence ratio | Not estimated | 0.91 (0.84, 0.99) | 8,024 |
| Aerobic-adherent subgroup, with additional aerobic-volume adjustment | Prevalence ratio | Not estimated | 0.92 (0.85, 1.00) | 8,024 |
| Inverse-probability weighting for complete-case inclusion | Prevalence ratio | 0.62 (0.58, 0.66) | 0.86 (0.79, 0.93) | 22,344 |

Note. Estimates are adjusted prevalence ratios with 95% confidence intervals. Models used the primary adjustment set unless otherwise specified. The reduced-adjustment model excluded body mass index and chronic conditions. Aerobic-volume analyses were restricted to 8,024 adults with valid reconstructed aerobic activity minutes who met the aerobic guideline according to the official activity classification. Survey-weighted median weekly aerobic-equivalent minutes (interquartile range) were 315 (210–540) in the aerobic-only group and 420 (240–630) in the both-guidelines group. Fifty-four respondents classified as meeting the aerobic guideline had reconstructed values below 150 minutes per week, including four with zero minutes; the official classification was retained. Inverse-probability weights for complete-case inclusion were estimated among all 24,319 arthritis-eligible respondents and applied to the 22,344 complete cases. Uncertainty was estimated using 400 stratified primary sampling unit bootstrap replicates that refitted the inclusion and outcome models.

**Supplementary Table S5. Year-specific associations, effect modification, severe mobility limitation, and exploratory functional outcomes by leisure-time activity guideline adherence among adults with doctor-diagnosed arthritis in the United States, 2020, 2022, and 2024.**

**Panel A. Year-specific associations and global survey-year interaction**

| **Survey year** | **Activity-pattern comparison** | **Adjusted prevalence ratio (95% confidence interval)** | **Model sample size** |
| --- | --- | --- | --- |
| 2020 | Both guidelines met vs neither guideline met | 0.67 (0.60, 0.76) | 7,644 |
| 2020 | Muscle-strengthening only vs neither guideline met | 0.90 (0.81, 1.01) | 7,644 |
| 2020 | Aerobic only vs neither guideline met | 0.73 (0.68, 0.80) | 7,644 |
| 2020 | Both guidelines met vs aerobic only | 0.92 (0.81, 1.05) | 7,644 |
| 2022 | Both guidelines met vs neither guideline met | 0.63 (0.56, 0.71) | 6,675 |
| 2022 | Muscle-strengthening only vs neither guideline met | 0.91 (0.82, 1.01) | 6,675 |
| 2022 | Aerobic only vs neither guideline met | 0.70 (0.64, 0.76) | 6,675 |
| 2022 | Both guidelines met vs aerobic only | 0.90 (0.79, 1.03) | 6,675 |
| 2024 | Both guidelines met vs neither guideline met | 0.57 (0.50, 0.65) | 8,025 |
| 2024 | Muscle-strengthening only vs neither guideline met | 1.00 (0.91, 1.10) | 8,025 |
| 2024 | Aerobic only vs neither guideline met | 0.74 (0.68, 0.80) | 8,025 |
| 2024 | Both guidelines met vs aerobic only | 0.77 (0.66, 0.89) | 8,025 |

**Panel B. Exploratory effect modification for both guidelines met versus neither guideline met**

| **Effect modifier** | **Stratum** | **Adjusted prevalence ratio (95% confidence interval)** | **Model sample size** |
| --- | --- | --- | --- |
| Sex | Male | 0.62 (0.55, 0.70) | 8,692 |
| Sex | Female | 0.63 (0.57, 0.68) | 13,652 |
| Age group | 18–64 years | 0.61 (0.55, 0.69) | 9,127 |
| Age group | 65 years or older | 0.63 (0.58, 0.69) | 13,217 |
| Obesity status | Without obesity | 0.59 (0.53, 0.65) | 13,144 |
| Obesity status | With obesity | 0.69 (0.62, 0.76) | 9,200 |

**Panel C. Severe mobility limitation**

| **Outcome** | **Activity-pattern comparison** | **Adjusted prevalence ratio (95% confidence interval)** | **Model sample size** |
| --- | --- | --- | --- |
| Severe mobility limitation | Both guidelines met vs neither guideline met | 0.41 (0.34, 0.49) | 22,344 |
| Severe mobility limitation | Muscle-strengthening only vs neither guideline met | 0.98 (0.86, 1.12) | 22,344 |
| Severe mobility limitation | Aerobic only vs neither guideline met | 0.44 (0.39, 0.50) | 22,344 |
| Severe mobility limitation | Both guidelines met vs aerobic only | 0.92 (0.75, 1.13) | 22,344 |

**Panel D. Proportional-odds assumption diagnostic for the four-level mobility outcome**

| **Outcome** | **Diagnostic** | **Test statistic** | **Model sample size** | **P value** |
| --- | --- | --- | --- | --- |
| Four-level mobility difficulty | Design-based joint Wald test comparing activity-pattern coefficients across three cumulative thresholds | F(6, 1586) = 5.22 | 22,344 | <0.001 |

Evidence against proportional-odds assumption; common odds ratios are not interpreted.

**Panel E. Prespecified exploratory functional outcomes**

| **Exploratory outcome** | **Activity-pattern comparison** | **Adjusted prevalence ratio (95% confidence interval)** | **Model sample size** |
| --- | --- | --- | --- |
| Difficulty doing errands alone | Both guidelines met vs neither guideline met | 0.37 (0.31, 0.44) | 22,346 |
| Difficulty doing errands alone | Muscle-strengthening only vs neither guideline met | 0.92 (0.81, 1.04) | 22,346 |
| Difficulty doing errands alone | Aerobic only vs neither guideline met | 0.49 (0.43, 0.55) | 22,346 |
| Difficulty doing errands alone | Both guidelines met vs aerobic only | 0.75 (0.62, 0.92) | 22,346 |

**Panel E (continued). Prespecified exploratory functional outcomes**

| **Exploratory outcome** | **Activity-pattern comparison** | **Adjusted prevalence ratio (95% confidence interval)** | **Model sample size** |
| --- | --- | --- | --- |
| Difficulty participating in social activities | Both guidelines met vs neither guideline met | 0.51 (0.44, 0.59) | 22,329 |
| Difficulty participating in social activities | Muscle-strengthening only vs neither guideline met | 0.96 (0.86, 1.08) | 22,329 |
| Difficulty participating in social activities | Aerobic only vs neither guideline met | 0.53 (0.48, 0.58) | 22,329 |
| Difficulty participating in social activities | Both guidelines met vs aerobic only | 0.97 (0.82, 1.14) | 22,329 |

**Notes.**

Panel A contains year-specific associations. The activity-pattern-by-year joint Wald test is reported in Results, Section 3.3.

Panel B contains exploratory subgroup associations. The global joint Wald tests are reported in Results, Section 3.3.

Panel C presents severe mobility limitation, defined as a lot of difficulty or inability to walk or climb steps.

Panel D presents the joint Wald test of activity-pattern coefficients across three cumulative thresholds. The proportional-odds assumption was not supported; common odds ratios are not interpreted.

Panel E presents exploratory outcomes for errands and social participation; outcome-specific complete-case sample sizes differ.

Association estimates are adjusted prevalence ratios and 95% confidence intervals from survey-weighted modified Poisson models. Interaction and diagnostic P values are unadjusted.
